# Unsupervised phenotyping of sepsis using nonnegative matrix factorization of temporal trends from a multivariate panel of physiological measurements

**DOI:** 10.1186/s12911-021-01460-7

**Published:** 2021-04-09

**Authors:** Menghan Ding, Yuan Luo

**Affiliations:** Department of Preventive Medicine, Feinberg School of Medicine, Northwestern University, Chicago, IL 60611 USA

**Keywords:** Sepsis, Phenotyping, Physiological measurements, Intensive care unit, Unsupervised learning, Clustering, Nonnegative matrix factorization, Frequent subgraph mining, Gradient boosting machine

## Abstract

**Background:**

Sepsis is a highly lethal and heterogeneous disease. Utilization of an unsupervised method may identify novel clinical phenotypes that lead to targeted therapies and improved care.

**Methods:**

Our objective was to derive clinically relevant sepsis phenotypes from a multivariate panel of physiological data using subgraph-augmented nonnegative matrix factorization. We utilized data from the Medical Information Mart for Intensive Care III database of patients who were admitted to the intensive care unit with sepsis. The extracted data contained patient demographics, physiological records, sequential organ failure assessment scores, and comorbidities. We applied frequent subgraph mining to extract subgraphs from physiological time series and performed nonnegative matrix factorization over the subgraphs to derive patient clusters as phenotypes. Finally, we profiled these phenotypes based on demographics, physiological patterns, disease trajectories, comorbidities and outcomes, and performed functional validation of their clinical implications.

**Results:**

We analyzed a cohort of 5782 patients, derived three novel phenotypes of distinct clinical characteristics and demonstrated their prognostic implications on patient outcome. Subgroup 1 included relatively less severe/deadly patients (30-day mortality, 17%) and was the smallest-in-size group (n = 1218, 21%). It was characterized by old age (mean age, 73 years), a male majority (male-to-female ratio, 59-to-41), and complex chronic conditions. Subgroup 2 included the most severe/deadliest patients (30-day mortality, 28%) and was the second-in-size group (n = 2036, 35%). It was characterized by a male majority (male-to-female ratio, 60-to-40), severe organ dysfunction or failure compounded by a wide range of comorbidities, and uniquely high incidences of coagulopathy and liver disease. Subgroup 3 included the least severe/deadly patients (30-day mortality, 10%) and was the largest group (n = 2528, 44%). It was characterized by low age (mean age, 60 years), a balanced gender ratio (male-to-female ratio, 50-to-50), the least complicated conditions, and a uniquely high incidence of neurologic disease. These phenotypes were validated to be prognostic factors of mortality for sepsis patients.

**Conclusions:**

Our results suggest that these phenotypes can be used to develop targeted therapies based on phenotypic heterogeneity and algorithms designed for monitoring, validating and intervening clinical decisions for sepsis patients.

## Background

Sepsis is a major public health challenge, both in the United States and worldwide [1]. It is one of the major diagnoses in Intensive care unit (ICU) patients and a leading cause of death and cost overruns [2–4].

Recent studies have estimated that approximately 1.7 million adults suffer from sepsis, and sepsis incidence has been steadily rising each year in the United States. Sepsis accounts for 30–50% of deaths among all hospitalizations, with an average cost of care over $50,000 per patient [5–8]. Although significant resources have been devoted to sepsis management, these allocations have not resulted in therapies that effectively lower the incidence or mortality of the disease [5]. Existing therapies, such as early goal-directed therapy (EGDT), focus on treating patients with severe sepsis or septic shock, who make up approximately 10% of all sepsis cases, but standardized and validated therapies are underdeveloped for the remaining majority of patients with less severe sepsis [8–10]. Sepsis is a complex heterogeneous syndrome that manifests in patients with diverse demographic profiles, correlated clinical variables, and underlying medical conditions, increasing the difficulty of developing targeted therapies.

The new Sepsis-3 definition developed at the Third International Consensus Conference in 2016 emphasizes the heterogeneity of sepsis. Sepsis-3 adopts the Sequential organ failure assessment score (SOFA) score in the diagnostic criteria, which consists of a panel of physiological variables. It deviates from the concept of staging in sepsis progression from systemic inflammatory response syndrome (SIRS) to severe sepsis to septic shock that was developed based on outcome stratification of mortality and severity. It removes the term severe sepsis and identifies septic shock as a subset of sepsis rather than the end stage [11]. A recent study also demonstrated that the updated Sepsis-3 definition identifies a 17–40% larger cohort compared to previous sepsis definitions [12]. Hence, previously undiscovered phenotypes may be derived from Sepsis-3 cohort that characterize on different combinations of heterogeneous features, and therefore provide implications for effective targeted therapies and improved patient management in the setting of real-time monitoring and timely interventions [13].

In this study, we aimed to identify and characterize novel phenotypes of sepsis for therapeutic and prognostic use based on temporal trends derived from a multivariate panel of physiological variables. We first collected records of physiological measurements within the first 3 days after the ICU admission, from which we derived frequent subgraphs to describe each ICU stay. We then applied nonnegative matrix factorization using frequent subgraphs as features and extracted trends of physiological measurements. As a result, patients were clustered into three subgroups based on their representative trend group. Finally, we demonstrated the clinical relevance of the identified patient clusters by characterizing them based on patient demographics and outcomes, distinguishing physiological trend groups, SOFA score trends and comorbidities, and that these patient clusters were prognostic factors of patient mortality.

## Methods

### Data

The data for this study was collected from the Medical information mart for intensive care III (MIMIC-III) database, an openly available dataset developed by the MIT Lab for Computational Physiology. It contains deidentified electronic health records (EHRs) from + 60,000 ICU stays at the Beth Israel Deaconess Medical Center (BIDMC, Boston, MA) between 2001 and 2012 [14].

We retrospectively defined the cohort for this study as patients whose sepsis onset was approximately aligned with their ICU admission using the new Sepsis-3 criteria from the MIMIC-III database. Since the MIMIC III database contains only data of the completed ICU stay and within 24 h after ICU discharge and the measurement period for physiological variables in our study was defined as the first 3 days after ICU admission, we chose to focus on patients suspected of developing sepsis within ± 24 h of their ICU admission to ensure that the physiological records extracted coincided with and particularly captured the early-to-mid phase of the sepsis trajectory. We referenced a recent study of a comparative analysis of sepsis identification algorithms including Sepsis-3 criteria using the MIMIC-III database [12]. We obtained their codes and adapted them for our use of identifying Sepsis-3 patients, and calculating Elixhauser Comorbidity Index and maximum daily SOFA scores over the first 7 days after ICU admission.

A total of 5782 patients were selected based on inclusion criteria. We then examined the value distribution of the physiological variables by visualizing them through histograms and referencing reference ranges, and removed records with values outside the measurable range as outliers (e.g., a respiratory rate value of 2,355,555 breaths per minute). The clean dataset after outlier handling exhibited distributions that were clinically justified.

### Candidate physiological variables

We selected 34 candidate physiological variables based on their association with sepsis onset and outcome and their common inclusion in predictive models for sepsis and overall mortalities in the ICU [15–18]. These 34 candidate physiological variables are described in Table 1.

**Table 1:** Candidate physiological variables with mean and standard deviation

Physiological variable	Mean	SD	Physiological variable	Mean	SD
Heart rate (bpm)	88.4	19.0	Platelet count (K/uL)	215.3	147.6
Respiration rate (insp/min)	20.4	6.2	Partial prothrombin time (sec)	40.4	17.1
Glasgow coma scale motor	5.1	1.5	International normalized ratio	1.5	0.6
Mean arterial blood pressure (mmHg)	79.2	18.2	Blood urea nitrogen (mg/dL)	32.8	25.8
Diastolic blood pressure (mmHg)	61.7	14.7	Blood serum creatinine (mg/dL)	1.5	1.3
Systolic blood pressure (mmHg)	121.6	23.7	Blood total bilirubin (mg/dL)	3.0	4.3
Urine output (mL)	120.7	114.6	Blood direct bilirubin (mg/dL)	4.4	5.4
Temperature (Celsius)	37.1	0.9	Aspartate aminotransferase (IU/L)	61.7	43.1
Blood oxygen saturation (%)	97.0	3.2	Base excess (mEq/L)	− 0.1	5.6
Fraction of inspired O_2_ (%)	47.5	14.8	Glucose (mg/dL)	136.7	51.2
Partial pressure of oxygen (mmHg)	99.4	34.0	Chloride (mEq/L)	105.2	6.9
Pao_2_/FiO_2_ ratio	208.2	110.5	Bicarbonate (mEq/L)	24.7	5.4
White blood cell count (K/uL)	12.1	6.9	Lactate (mmol/L)	2.3	1.6
Hemoglobin (g/dl)	10.0	1.8	Blood albumin (g/dL)	2.8	0.6
Hematocrit (%)	29.6	5.2	Carbon dioxide (mEq/L)	26.0	6.3
Ph (unit)	7.4	0.1	Blood serum potassium (mEq/L)	4.1	0.7
Magnesium (mg/dL)	2.1	0.4	Blood sodium (mEq/L)	139.3	5.8

*SD* standard deviation

### Time series graphs

Existing studies that involve a panel of physiological variables often use numerical measurements collected from a single time point to define or interpret a clinical event. In reality, a clinical event is captured in and described by a series of fluctuating vital signs and laboratory test results with covariations over time. Therefore, we used time series graphs to represent the panel of physiological data in our study, as graphs are more expressive and informative in representing the trends and variations in data over time.

Time series of physiological data were extracted from the data describing the first 3 days after ICU admission. Since physiological variables are often irregularly and sparsely recorded, discretization of a physiological time series is important to obtain error-mitigated and uniformly paced time series graphs [18]. We used Pandas linear interpolation implementation to discretize the time series along the time axis across uniform time intervals with imputation for time intervals that had no physiological values recorded based on the rest populated time intervals for the same patient. We performed time series discretization for two different time intervals (6- and 24-h intervals) and determined the optimal length of the time interval to be 6 h in the hyperparameter tuning step for nonnegative matrix factorization, based on the stability of the clustering results and the distinctiveness of the frequent subgraph distributions within each cluster. We also standardized the interpolated values for each physiological variable into z-scores with rounding to discretize the time series along the measurement axis. After discretization, we generated time series graphs by concatenating physiological z-scores in the sequence of time intervals into a tuple in python, a collection that is ordered and unchangeable. The set of time series graphs encompassing all patients’ ICU stays formed a corpus, and 34 corpora corresponding to 34 candidate physiological variables were formed.

### Frequent subgraph mining

Frequent subgraph mining is a pattern mining technique used to discover patterns as subgraphs in a graph corpus (a set of graphs) based on a certain frequency threshold (minimum support threshold). This technique effectively identifies frequent patterns (also referred to as temporal trends) in time series graphs, removes noise in the data for modeling and interpretation, and has been successfully applied to studies for phenotyping and predicting outcomes in multiple diseases [18, 19].

We performed frequent subgraph mining on the corpora of 34 physiological variables over 5 different choices of the minimum support threshold (5, 15, 25, 50, and 100) and determined the optimal minimum support threshold to be 5 in the hyperparameter tuning step for nonnegative matrix factorization, again based on the stability of the clustering results and the distinctiveness of the frequent subgraph distributions within each cluster. We limited the size of a subgraph to a minimum of 2 nodes and a maximum of 6 nodes to ensure that the subgraphs were interpretable and that distinctive patterns were easy to identify. A total of 27,971 frequent subgraphs were identified at this step. A simple example of the application of the aforementioned frequent subgraph mining algorithm to mine subgraphs from the graph corpus of one patient and one physiological variable is presented in “Appendix 1”. We then applied subgraph isomorphism removal at the patient level such that when a larger subgraph was presented, counts of smaller subgraphs would be set to 0 for that particular patient’s case [18]. 5600 subgraphs were thus removed. The final matrix of patient-subgraph counts contained 22,371 subgraphs.

### Subgraph augmented NMF

Unsupervised clustering methods capture the inherent structure and correlation within a population and identify natural clusters with significant within-cluster similarities and between-cluster differences. Nonnegative matrix factorization is one of the unsupervised clustering methods that has been applied to effectively derive patient subgroups in multiple diseases, particularly because of the good interpretability in its result due to nonnegativity constraints [19–21]. In our study, we applied non-negative matrix factorization (NMF) over a patient-subgraph count matrix to derive temporal trend groups of covariations and patient subgroups such that the physiological progression of each patient subgroup was described by the corresponding temporal trend group.

The patient-subgraph count matrix was split into training and testing sets at an 80:20 ratio stratified by mortality. We profiled both sets with clinical variable distributions to confirm that the testing set was representative of the training set. We then fit the NMF model by performing hyperparameter tuning on the training set over the hyperparameters, including time interval, minimum support threshold, and number of components. We determined the optimal combination of hyperparameters to be a time interval equal to 6 h, a minimum support threshold equal to 5, and the number of components equal to 3, using cophenetic correlation and distinctiveness of frequent subgraph distributions within each cluster [22]. We validated our choice of optimal NMF model configuration by refitting the optimal model configuration on the testing set and comparing the cophenetic correlation pattern and subgraphs’ weight distribution between the training set and the testing set, confirming that the model performance of the training set was recapitulated in the testing set. We used cophenetic correlation implemented in Nimfa and projected gradient NMF implemented in Scikit-learn [23–25].

### Identifying Sepsis-3 subgroups

We next applied subgraph-augmented nonnegative matrix factorization with the optimal configuration determined in the hyperparameter tuning step over the entire Sepsis-3 cohort and separated the cohort into 3 distinct subgroups based on the model output.

The NMF model outputs were two lower ranked matrices, the patient group coefficient matrix (5782 × 3) and the trend group coefficient matrix (3 × 22,371), both with nonnegative values decomposed from the input matrix of patient-subgraph counts. The trend group coefficient matrix served to form three trend groups that were weighted composites of the 22,371 frequent subgraphs such that each trend group encompassed frequent physiological trends observed in a corresponding patient subgroup. The patient group coefficient matrix then served to assign patient membership to the corresponding trend group where patients had the highest group coefficient. This successfully separated the Sepsis-3 patients into three subgroups, each associated with and described by its corresponding trend group.

To further summarize each trend group with dominant subgraphs from the 22,371 subgraphs extracted, subgraphs that were either ranked in the top 100 or had a value greater than 1 in terms of group coefficients were selected as representative subgraphs to summarize each trend group. Three sets of representative subgraphs were thus selected to summarize and represent their corresponding trend group and the associated patient subgroup. To validate the three sets of selected subgraphs’ representativeness of their corresponding patient subgroups, we trained a gradient boosting machine for multiclass classification of patient group membership over the three sets of selected subgraphs combined and achieved overall 91.7% accuracy in the testing set from a refreshed train-test split.

We then analyzed outcome distributions and underlying clinical patterns in the three patient subgroups identified above and performed functional validations to assess the prognostic implications of patient group membership on mortality. To provide more clarity into the process of NMF model construction, Sepsis-3 subgroup identification, and the associated functional validations, we included a flow diagram that demonstrates these processes in “Appendix 2”.

## Results

The Sepsis-3 patients were separated into 3 distinct subgroups based on their physiological trends within the first 72 h after ICU admission. These three subgroups exhibited distinct clinical characteristics in terms of patient demographics and outcomes, physiological patterns, disease trajectories and comorbidities and were prognostic factors of patient mortality.

### Demographics and outcomes

Patients in the Sepsis-3 subgroups had variable demographic characteristics and distinct outcomes, which are described in Table 2 and below:

- *Subgroup 1* oldest (73.1 years), most overweight or obese (84.3 kg); high in male (59.0%), Elixhauser index (3.5), 30-day mortality (17.0%) and in-hospital mortality (12.0%)
- *Subgroup 2* younger (67.9 years), less overweight or obese (83.0 kg); highest in male (60.3%), Elixhauser index (5.7), day-1 SOFA score (8.7), length of stay (6.2 days), 30-day mortality (28.4%) and in-hospital mortality (24.8%)
- *Subgroup 3* youngest (59.9 years), least overweight or obese (79.4 kg), balanced sex (50-to-50); low in

**Table 2:** Demographics and outcome

	Subgroup 1	Subgroup 2	Subgroup 3	Sepsis-3 cohort
Group size	1218	2036	2528	5782
Age, mean (year) ± SD	73.12 ± 14.56	67.91 ± 16.27	59.92 ± 18.24	65.52 ± 17.64
Gender (%)				
Male	59.03%	60.27%	50.44%	55.50%
Female	40.97%	39.73%	49.56%	44.30%
Weight, mean (kg) ± SD	84.25 ± 35.08	83.01 ± 24.32	79.37 ± 25.54	81.68 ± 27.56
BMI, mean (kg/m^2^) ± SD	29.36 ±	29.48 ±	28.36 ±	29.01 ± 8.73
BMI, Strata				
Overweight	32.50%	32.00%	33.14%	32.56%
Underweight	1.88%	2.72%	3.78%	2.96%
Obese	37.50%	37.61%	30.67%	34.81%
Healthy weight	28.12%	27.67%	32.40%	29.66%
ICU LOS, mean (day) ± SD	3.14 ± 4.67	6.23 ± 7.10	4.21 ± 5.52	4.69 ± 6.09
Elixhauser index, Mean ± SD	3.53 ± 6.90	5.65 ± 7.12	2.37 ± 6.59	3.77 ± 6.99
Ethnicity (%)				
Asian	2.38%	2.95%	3.56%	3.10%
Black	9.61%	8.64%	8.23%	8.66%
White	76.68%	72.15%	71.08%	72.64%
Hispanic	1.40%	3.09%	4.27%	3.25%
Other	9.93%	13.16%	12.86%	12.35%
Day-1 SOFA score, Mean ± SD	6.51 ± 2.60	8.65 ± 3.38	6.50 ± 2.53	7.29 ± 3.07
30-Day mortality (%)	17.00%	28.39%	10.13%	18.00%
In-hospital mortality (%)	11.99%	24.80%	7.32%	14.46%

*SD* standard deviation

Elixhauser index (2.4), 30-day mortality (10.1%) and in-hospital mortality (7.3%).

Statements made above for significant (or non-significant) subgroup characteristics were consistent with the results of the hypothesis tests performed to determine differences in the means between subgroups for these characteristics at the 0.05 significance level (“Appendix 3”).

The Sepsis-3 cohort consisted of older (65.5 years), overweight or obese (67.4%), male (55.5%) and white (72.6%) patients in majority, with an overall 30-day mortality rate of 18%. Our study essentially separated the cohort into three distinct subgroups such that subgroup 3 consisted of the least sick and fewest elderly patients marked by the lowest mortality, while subgroup 2 and subgroup 1 further separated the sicker patients into the sickest group with most severe conditions marked by the highest mortality, and an older but less sick group with more chronic conditions marked by lower mortality.

### Representative subgraphs

Utilizing nonnegative matrix factorization, we identified three distinct trend groups from physiological time series graphs that were subsequently used to cluster patients and describe patient subgroups. We characterized each trend group with representative subgraphs that were either ranked in the top 100 or had a value greater than 1 in terms of group coefficients. A total of 166 unique subgraphs were chosen, out of which 67 exclusively described one subgroup, indicating that the identified subgroups were distinctively characterized by these subgraphs. To validate that the three sets of selected subgraphs were sufficiently representative of the three corresponding patient subgroups, we trained a gradient boosting machine classifying patient group membership using the three sets of representative subgraphs combined and achieved overall 91.7% accuracy in the testing set from a refreshed train-test split. Additionally, the subgroup-wise precision, recall, f-score and area under the curve (AUC) are shown in Table 3, and the ROC curves from the testing set are included in Appendices 4, 5, and 6.

**Table 3:** Gradient boosting machine error metrics for patient group membership classification on frequent subgraphs

Measure	Split	Subgroup		
		1	2	3
Precision	Train	0.987	0.981	0.988
	Test	0.917	0.889	0.939
Recall	Train	0.978	0.984	0.990
	Test	0.835	0.921	0.951
F-score	Train	0.982	0.983	0.989
	Test	0.874	0.905	0.945
AUC	Train	0.954	0.964	0.977
	Test	0.891	0.914	0.944

**Fig. 1:**
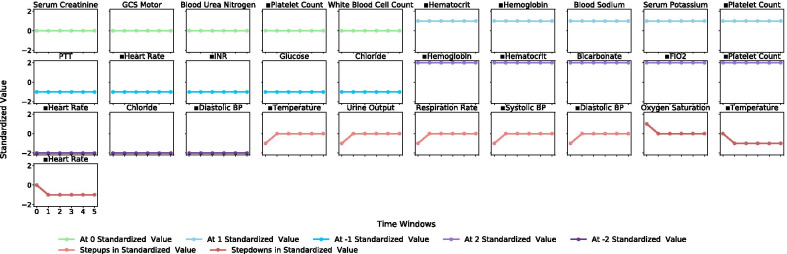
Subgroup 1 trend group selected from representative frequent subgraphs of standardized physiological variable values over measurement period of six time windows

**Fig. 2:**
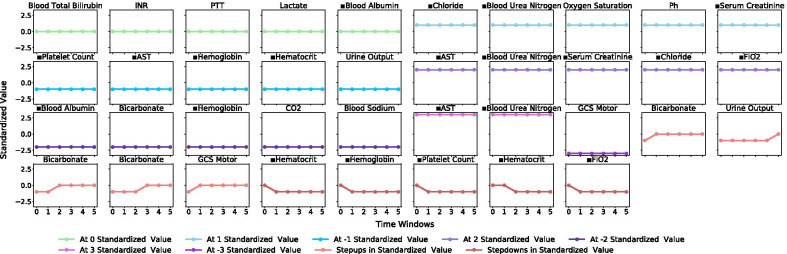
Subgroup 2 trend group selected from representative frequent subgraphs of standardized physiological variable values over measurement period of six time windows

**Fig. 3:**
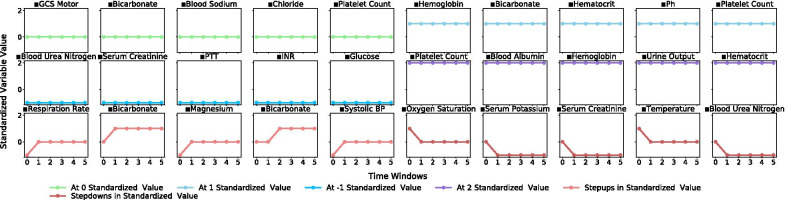
Subgroup 3 trend group selected from representative frequent subgraphs of standardized physiological variable values over measurement period of six time windows

Distinct clinical patterns that indicate underlying medical conditions in each patient subgroup were observed in the representative subgraphs. Subgroup 1 (Fig. 1) was subjected to high cardiovascular and respiratory dysfunction marked by low to decreasing heart rate, blood pressure and temperature and a high fraction of inspired O_2_ (FiO_2_), while incurring low organ dysfunction, inflammation and coagulopathy, as indicted by high platelet counts, hematocrit and hemoglobin levels, and a low international normalized ratio (INR) and partial prothrombin time (PTT). Subgroup 2 (Fig. 2) exhibited patterns of severe renal, hepatic and respiratory dysfunction marked by low platelet counts and high administration of aspartate aminotransferase (AST), high blood serum creatinine, blood urea nitrogen and chloride, low blood albumin, and a high fraction of inspired O_2_, coupled with high inflammation marked by low to decreasing hemoglobin and hematocrit levels. Subgroup 3 (Fig. 3) was described primarily by improving or stabilizing physiological patterns that indicated a comparatively better and quickly improving medical condition. The referenced subgraphs are marked with ■ in the figures. These patterns also manifested in corresponding SOFA score trends and comorbidity distributions of the three subgroups.

Patient subgroups containing trends that indicated a general progression to the better and stabilized states (e.g., Subgroup 1 in Fig. 1, Subgroup 3 in Fig. 3) were often associated with positive patient outcome. Patient subgroups containing trends that indicated a general progression to the worse state were often associated with negative patient outcome (e.g., Subgroup 2 in Fig. 2). To validate this association, we trained a gradient boosting machine modeling mortality using the three sets of selected subgraphs combined, representing the three patient subgroups, and achieved 86.3% accuracy and 68.1% AUC in the testing set, again from a refreshed train-test split. Additional details and evaluation metrics are discussed and shown under “Implications on patient outcome” section.

### 7-Day SOFA score trend

**Fig. 4:**
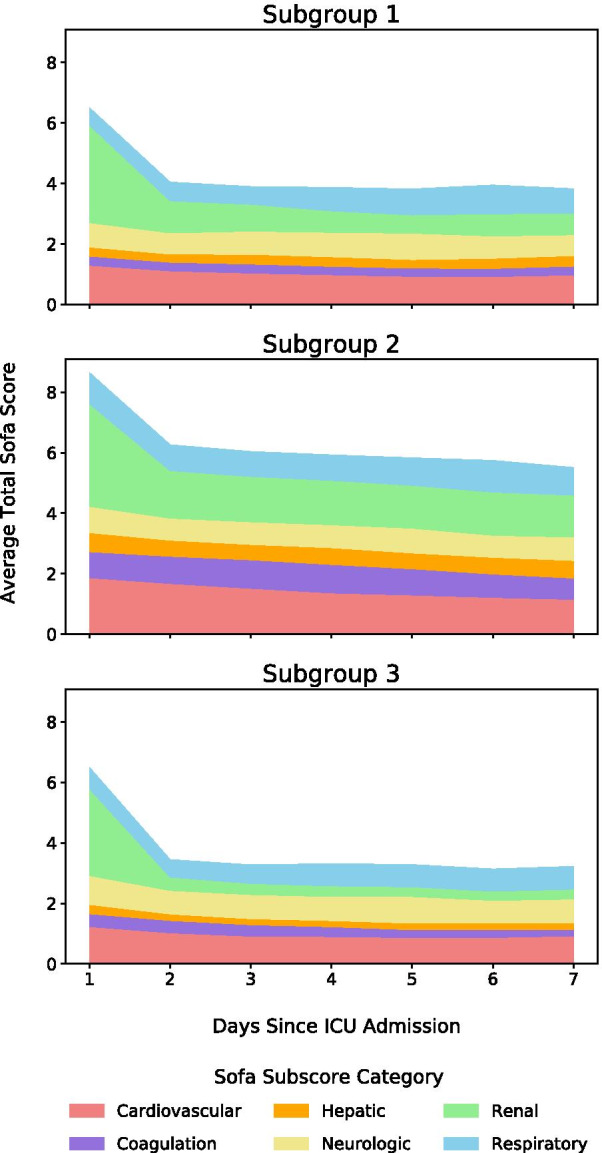
7-Day sofa score charts

Patients in the Sepsis-3 subgroups had variable disease trajectories over their ICU stays. We captured disease trajectories of the subgroups by calculating and plotting the average daily SOFA scores for the first 7 days (Fig. 4).

Subgroup 1 had the second highest SOFA score on day 1 of ICU admission, which was similar to subgroup 3 but was differentiated by slow improvement from day 2 to day 7. Its SOFA score trajectory was largely driven by a high renal subscore followed by a high cardiovascular subscore. Subgroup 2 had the highest SOFA score on both day 1 and day 7, indicating the severity of organ dysfunction and underlying medical conditions in this group. Its SOFA score trajectory was also dominantly driven by high renal and cardiovascular subscores with a uniquely high hepatic subscore. Subgroup 3 exhibited the lowest SOFA scores and fastest improvement from day 1 to day 7, distinguished by a disproportionately high neurologic subscore. These patterns also manifested in the comorbidity distributions of the three subgroups.

### Comorbidities

Patients in the Sepsis-3 subgroups had variable underlying medical conditions characterized by their comorbidity distributions. We utilized the method developed in the Elixhauser Comorbidity Index and calculated the incidences of 30 categories of comorbidities for the three subgroups identified. We ranked the 30 categories by their cohort incidence in descending order and focused our analysis on 17 categories that had a minimal subgroup incidence of 10%, as shown in Table 4 [12, 26].

**Table 4:** Within-subgroup incidences of the top 17 comorbidities with a minimal 10% incidence by subgroup

Comorbidity category	Comorbidity incidence (%)		
	Subgroup 1	Subgroup 2	Subgroup 3
Hypertension	69.87	65.18	48.58
Fluid electrolyte imbalance	42.12	61.94	35.36
Cardiac arrhythmias	38.59	37.13	22.47
Congestive heart failure	32.35	30.60	14.95
Deficiency anemias	22.91	28.98	21.36
Diabetes uncomplicated	28.08	25.74	16.57
Chronic pulmonary	23.40	21.02	20.49
Renal failure	28.08	28.88	6.80
Coagulopathy	9.93	29.57	11.95
Neurologic disease	15.11	13.21	21.76
Hypothyroidism	15.35	13.26	10.72
Depression	11.66	12.97	14.04
Liver disease	6.16	17.44	9.49
Valvular disease	11.82	13.02	7.24
Alcohol abuse	6.08	10.61	12.86
Peripheral vascular	11.33	12.03	5.42
Pulmonary circulation	9.85	11.10	6.29

Comorbidity categories are sorted in descending order of their combined incidence in the sepsis cohort

Cardiovascular, renal, and pulmonary diseases were the dominant categories in the Sepsis-3 cohort, according to their combined incidence across subgroups. Specifically, cardiovascular diseases were dominant in subgroups 1 and 2, with incidence rates ranging from 22.9 to 69.9% (hypertension, fluid electrolyte imbalance, cardiac arrhythmias, congestive heart failure, anemia, and diabetes), followed by renal failure diseases, with incidence rates ranging from 28.1 to 28.9%. Chronic pulmonary diseases were a common category of high incidence across all three subgroups, with incidence rates ranging from 20.5 to 23.4%. Subgroups were also characterized by distinct underlying conditions on the following absolute or comparative bases:

- *Subgroup 1* low in coagulopathy (9.9%), liver disease (6.2%), and alcohol abuse (6.1%)
- *Subgroup 2* high in coagulopathy (29.6%), deficiency anemias (29.0%), liver disease (17.4%), and pulmonary circulation disease (11.1%)
- *Subgroup 3* low in all dominant categories, except for chronic pulmonary disease, deficiency anemias, and depression; high in neurologic disease (21.8%) and alcohol abuse (12.9%)

Statements made above for significant (or non-significant) subgroup characteristics were consistent with the results of the hypothesis tests performed to determine differences in the means between subgroups for these characteristics at the 0.05 significance level (“Appendix 3”).

Therefore, we further characterized these subgroups based on the heterogeneity observed in their comorbidity distributions. Subgroup 2, the sickest group with the highest mortality, was comprised of patients with severe organ dysfunction or failure multiplied by a wide range of comorbidities, whereas Subgroup 1, the less sick group with reduced mortality, consisted of patients with chronic conditions in cardiovascular and pulmonary categories. Subgroup 3 was our least sick group with the lowest mortality, distinguished by low comorbidities in general, except for a high incidence of neurologic diseases.

### Implications on patient outcome

The Sepsis-3 subgroups identified in this study had prognostic implication on clinical outcome. As was validated previously that the selected subgraphs were sufficiently representative of the three patient subgroups, we trained a gradient boosting machine to model patient mortality using the three sets of selected subgraphs combined to assess this implication. The model achieved 86.3% accuracy and 68.1% AUC in the testing set from a refreshed train-test split. Additional evaluation metrics, including precision, recall, and f-scores, are shown in Table 5, and the ROC curve from the testing set are included in “Appendix 7”.

**Table 5:** Gradient boosting machine error metrics for patient 30-day mortality model on frequent subgraphs representing patient subgroups

Measure	Train	Test
Accuracy	0.892	0.863
Precision	0.885	0.802
Recall	0.726	0.681
F-score	0.773	0.716
AUC	0.726	0.681

We then trained two other gradient boosting machines to model patient mortality using mean 7-day SOFA scores and the Elixhauser Comorbidity Index as benchmarks. The two preliminary models both underperformed the subgraph-based model with 83.7% accuracy and 56.0% AUC for the SOFA score-based model and 81.1% accuracy and 50.7% AUC for the comorbidity score-based model. Both sets of statistics were based on the testing sets of the refreshed train-test splits. Additional evaluation metrics, including precision, recall, and f-scores, are shown in Tables 6 and 7. These results validated that these patient subgroups were prognostic factors of patient mortality.

**Table 6:** Gradient boosting machine error metrics for patient 30-day mortality model on mean 7-day SOFA scores

Measure	Train	Test
Accuracy	0.846	0.837
Precision	0.809	0.759
Recall	0.583	0.560
F-score	0.602	0.566
AUC	0.583	0.560

**Table 7:** Gradient boosting machine error metrics for patient 30-day mortality model on Elixhauser comorbidity index

Measure	Train	Test
Accuracy	0.829	0.811
Precision	0.914	0.629
Recall	0.520	0.507
F-score	0.491	0.466
AUC	0.520	0.507

## Discussion

Several studies have been conducted by fellow researchers to identify subgroups in sepsis patients. Utilizing gene expression data, Sweeney et al. [27] performed two clustering analyses, K-means clustering and partitioning around medoids (PAM) clustering, of sepsis patients with bacterial infections. Three phenotypes were identified using combined results from both K-means and PAM clusters. Although Sweeney et al. applied additional limits to the cohort to include only bacteria-induced sepsis patients and exclude virus-induced sepsis patients and utilized a different type of dataset on gene expression rather than physiological measurements, our phenotyping results, upon comparison, had significant similarities to theirs. Our subgroup 2 was similar to their “coagulopathic” cluster, both characterized by old age, high mortality and clinical coagulopathy. Our subgroup 3 was similar to their “adaptive” cluster, both consisting of younger and less-sick patients with low mortality. More recently, Seymour et al. [28] conducted a K-means clustering analysis utilizing clinical data and derived four phenotypes from a robust cohort of 20,189 sepsis patients. We found similarities between our subgroup 3 and their α phenotype in terms of low age and mortality. In particular, low administration of vasopressors in the α phenotype agreed with the low cardiovascular diseases observed in subgroup 3. Our subgroup 2 was also similar to their δ, both exhibiting high mortality, male majority, and distinguished by liver dysfunction. Both of these previous studies essentially collected a single data point per time series to describe sepsis phenotypes, whereas our phenotypes were described using a set of physiological trends spanning the first 3 days at a minimal resolution of one data point for every 6 h.

We recognize a number of limitations in our study. First, our data were from only the ICU of one hospital in Boston, MA. Second, the data points used in our study were often irregularly and sparsely recorded. Missing values were imputed using linear approximation, which may have altered, obscured, or artificially boosted temporal trends that may have been, in fact, less significant. As future work, we plan to investigate more advanced imputation methods designed for multi-variable clinical time series. [29, 30] We also plan to validate this physiological subgraph-augmented NMF model with a robust cohort constructed from a geographically diverse set of locations to further assess the reproducibility and relevance of this novel phenotyping. Finally, our data source contains only records generated during the ICU stay and within 24 h after the ICU discharge, rather than the entire hospitalization or patient encounter. Data that capture early signs and development prior to disease onset tend to be missing or incomplete. Therefore, we designed our study to aim at deriving phenotypes that would characterize the disease trajectories of sepsis and provide prognostic implications for clinical outcomes rather than predictive implications for sepsis onset.

In this study, we demonstrated that the identified sepsis phenotypes displayed distinctive and clinically relevant characteristics and were decent prognostic factors of mortality. These phenotypes and associated findings could be useful with respect to developing targeted therapies and clinical interventions for sepsis. Efficiency in clinical trials to develop targeted therapies, which is often obfuscated by the inter-patient variability, could be improved by designing trials that selectively enroll patients with phenotypes as subgroups in the cohort, test different treatment approaches on these patient subgroups, and compare the results to identify specific treatments to which a given subgroup is more responsive [31]. The phenotypes identified in our study could serve as baseline phenotypes for designing clinical trials such that (1) patients are selectively enrolled into phenotypic subgroups in the sepsis cohort, (2) different therapies are tested, and responses are compared between patient subgroups, and (3) targeted therapies to which each of the subgroups is most responsive are identified. Intelligent alerts and validators in clinical decision support systems could be developed based on the representative subgraph groups, which describe the sepsis phenotypes, to effectively monitor sepsis patients. For example, alarms triggered by multiple physiological variables that trend toward a worsened state (e.g., subgroup 2 in Fig. 2) may provide more accurate information for physician and nurse to act on. Clinical decisions, such as discharging a patient from ICU, may be validated across multiple key physiological trends to mitigate the risk of premature discharge. Methods in our study may also be adapted to extract physiological trend groups as features to model patient outcomes and, given a more robust and complete data source, to predict disease onset to identify opportunities for early intervention.

## Conclusions

We identified three distinct phenotypes from patients with sepsis utilizing the novel algorithm subgraph-augmented nonnegative matrix factorization (SANMF) on temporal trends from a multivariate panel of physiological variables. These phenotypes were characterized by distinct demographics, physiological patterns, disease trajectories, and underlying comorbidities and were demonstrated to be prognostically relevant to clinical outcome. These findings could be leveraged to understand the heterogeneity in the progression and treatment effects of sepsis, and to develop targeted therapies to alternately address the impact of heterogeneity. Further research is needed to determine the feasibility of these initiatives.

## Figures and Tables

**Fig. 5:**
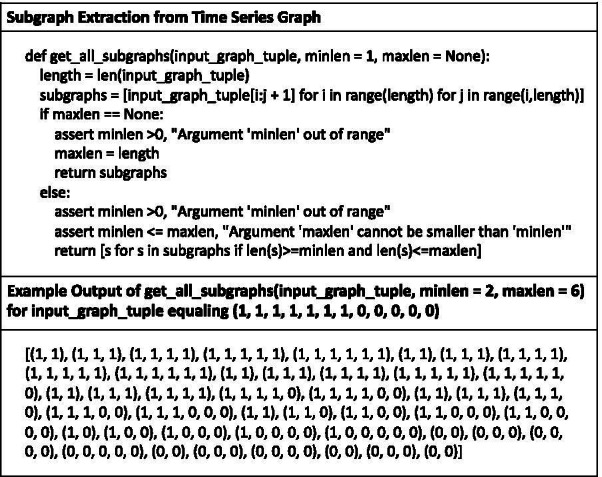
Subgraph extraction from time series graph

**Fig. 6:**
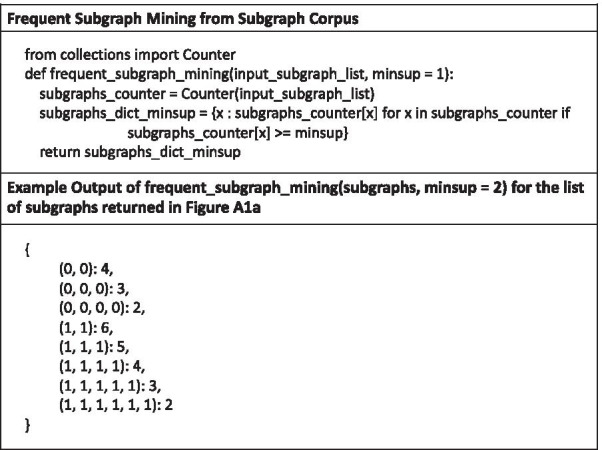
Frequent subgraph mining from subgraph corpus

**Fig. 7:**
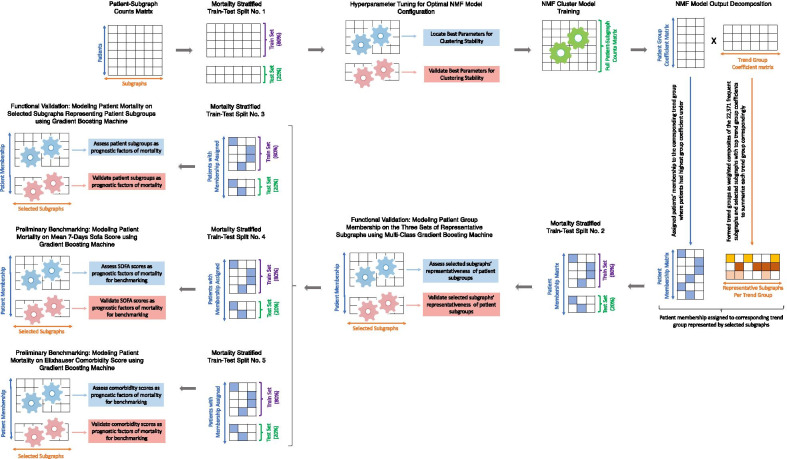
Flow diagram for NMF model construction, Sepsis-3 subgroup identification, and associated functional validations

**Fig. 8:**
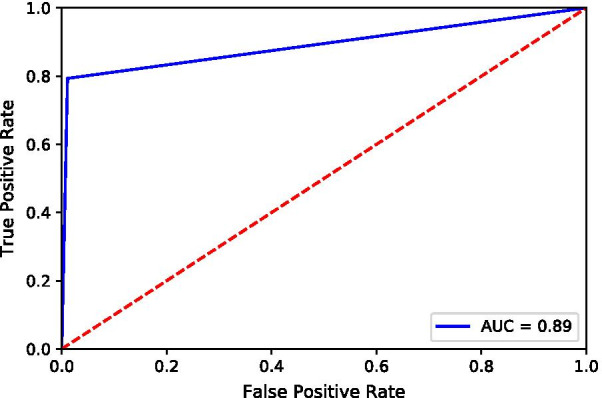
Gradient boosting machine ROC curve for patient group membership classification on frequent subgraphs—subgroup 1

**Fig. 9:**
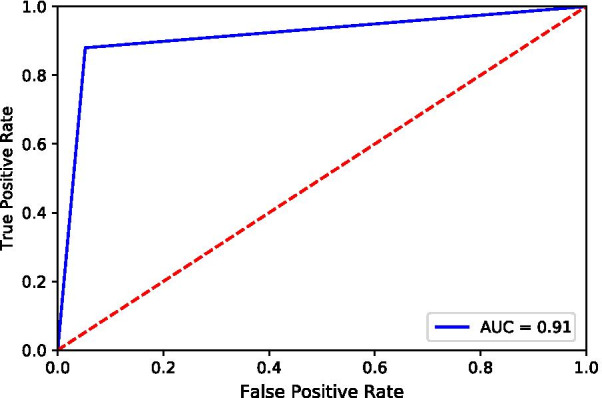
Gradient boosting machine ROC curve for patient group membership classification on frequent subgraphs—subgroup 2

**Fig. 10:**
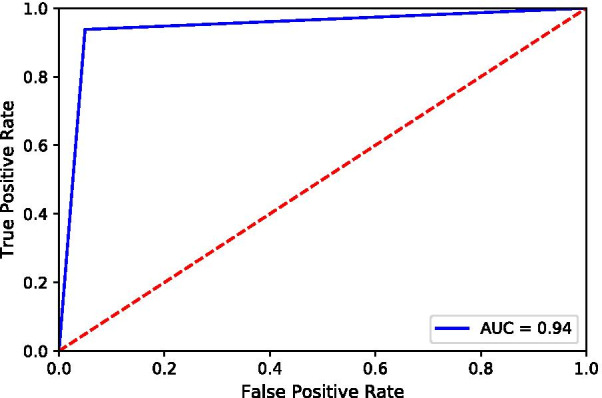
Gradient boosting machine ROC curve for patient group membership classification on frequent subgraphs—subgroup 3

**Fig. 11:**
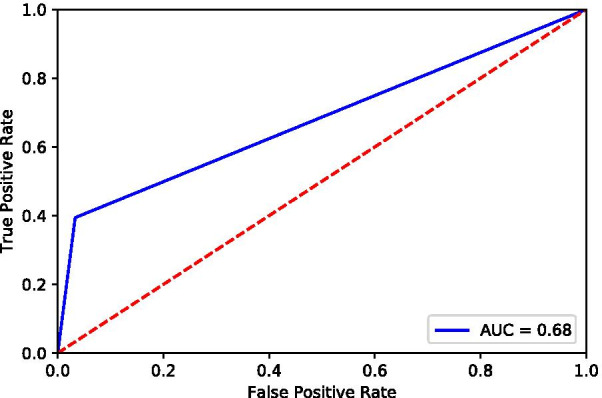
Gradient boosting machine ROC curve for patient mortality model on frequent subgraphs

## Data Availability

The datasets generated and/or analyzed during the current study are available on the MIMIC-III critical care database at https://mimic.physionet.org/.

## Appendix 1: Demonstration of key components in frequent subgraph mining implementation

We added a simple example of applying the frequent subgraph mining algorithm to mine subgraphs from the graph corpus of one patient and one physiological variable in “Appendix 1” to demonstrate the two key components “Subgraph Extraction from Time Series Graph” and “Frequent Subgraph Mining from Subgraph Corpus” in our python implementation of the frequent subgraph mining algorithm.

The component “Subgraph Extraction from Time Series Graph” is demonstrated in Fig. 5, where we included a simplified function called get_all_subgraphs(), which takes a graph corpus, the minimum length of subgraph as minlen, the maximum length of subgraph as maxlen as inputs and returns a list of subgraphs extracted from the input graph corpus. An example output is presented for the case in which the function is applied to a sample graph corpus of (1, 1, 1, 1, 1, 1, 1, 0, 0, 0, 0, 0) extracted from one patient and one physiological variable to extract all subgraphs with lengths between 2 and 6.

The component “Frequent Subgraph Mining from Subgraph Corpus” is demonstrated in Fig. 6, where we included a simplified function called frequent_subgraph_mining(), which takes a list of subgraphs and the minimum support threshold as inputs and returns a dictionary of all frequent subgraphs as keys and counts of occurrence as values. An example output is presented for the case in which the function was applied to the list of subgraphs extracted, as shown in Fig. 5, to obtain frequent subgraphs with a count of occurrence greater than or equal to 2.

## Appendix 2

See Fig. 7.

## Appendix 3

See Table 8.

**Table 8:** Two sample T Test results for distinguishing clinical characteristics by subgroup

Clinical characteristic	Mean (SD) within subgroup			Two-sample T Test t-statistic (significance level) for difference in means between subgroups		
	1	2	3	1 and 2	1 and 3	2 and 3
Gender (is male)	0.59 (0.49)	0.60 (0.49)	0.50 (0.50)	− 0.695 (*P* = 0.487)	4.954 (*P* < 1e−3)	6.664 (*P* < 1e−3)
Age	73.12 (14.56)	67.91 (16.27)	59.92 (18.24)	9.194 (*P* < 1e−3)	22.094 (*P* < 1e−3)	15.429 (*P* < 1e−3)
Weight (kg)	84.25 (35.08)	83.01 (24.32)	79.37 (25.54)	1.144 (*P* = 0.253)	4.659 (*P* < 1e−3)	4.661 (*P* < 1e−3)
Elixhauser index	3.53 (6.90)	5.65 (7.12)	2.37 (6.59)	− 8.334 (*P* < 1e−3)	4.957 (*P* < 1e−3)	16.136 (*P* < 1e−3)
Day-1 sofa score	6.51 (2.60)	8.65 (3.38)	6.50 (2.53)	− 17.575 (*P* < 1e−3)	0.104 (*P* = 0.917)	23.652 (*P* < 1e−3)
ICU LOS (day)	3.14 (4.67)	6.23 (7.10)	4.21 (5.52)	− 13.561 (*P* < 1e−3)	− 5.846 (*P* < 1e−3)	10.83 (*P* < 1e−3)
30-Day mortality	0.17 (0.38)	0.28 (0.45)	0.10 (0.30)	− 7.411 (*P* < 1e−3)	6.01 (*P* < 1e−3)	16.323 (*P* < 1e−3)
In-hospital mortality	0.12 (0.32)	0.26 (0.43)	0.07 (0.26)	− 8.95 (*P* < 1e−3)	4.729 (*P* < 1e−3)	16.893 (*P* < 1e−3)
Coagulopathy	0.10 (0.30)	0.30 (0.46)	0.12 (0.32)	− 13.388 (*P* < 1e−3)	− 1.823 (*P* = 0.068)	15.217 (*P* < 1e−3)
Liver disease	0.06 (0.24)	0.17 (0.38)	0.09 (0.29)	− 9.313 (*P* < 1e−3)	− 3.451 (*P* = 0.001)	7.975 (*P* < 1e−3)
Alcohol abuse	0.06 (0.24)	0.11 (0.31)	0.13 (0.33)	− 4.404 (*P* < 1e−3)	− 6.333 (*P* < 1e−3)	− 2.335 (*P* = 0.020)
Pulmonary circulation	0.10 (0.30)	0.11 (0.31)	0.06 (0.24)	− 1.117 (*P* = 0.264)	3.897 (*P* < 1e−3)	5.833 (*P* < 1e−3)
Neurological disease	0.15 (0.36)	0.13 (0.34)	0.22 (0.41)	1.511 (*P* = 0.131)	− 4.817 (*P* < 1e−3)	− 7.522 (*P* < 1e−3)
Chronic pulmonary	0.06 (0.24)	0.11 (0.31)	0.13 (0.33)	1.587 (*P* = 0.113)	2.033 (*P* = 0.042)	0.440 (*P* = 0.660)
Hypertension	0.70 (0.46)	0.65 (0.48)	0.49 (0.50)	2.756 (*P* = 0.006)	12.536 (*P* < 1e−3)	11.386 (*P* < 1e−3)
Fluid electrolyte imbalance	0.42 (0.49)	0.62 (0.49)	0.35 (0.48)	− 11.192 (*P* < 1e−3)	4.006 (*P* < 1e−3)	18.530 (*P* < 1e−3)
Cardiac arrhythmias	0.39 (0.49)	0.37 (0.48)	0.22 (0.42)	0.829 (*P* = 0.407)	10.473 (*P* < 1e−3)	10.991 (*P* < 1e−3)
Congestive heart failure	0.32 (0.47)	0.31 (0.46)	0.15 (0.36)	1.041 (*P* = 0.298)	12.585 (*P* < 1e−3)	12.926 (*P* < 1e−3)
Deficiency anemias	0.23 (0.42)	0.29 (0.45)	0.21 (0.41)	− 3.796 (*P* = 0.000)	1.072 (*P* = 0.284)	5.949 (*P* < 1e−3)
Diabetes uncomplicated	0.28 (0.45)	0.26 (0.44)	0.17 (0.37)	1.463 (*P* = 0.144)	8.270 (*P* < 1e−3)	7.646 (*P* < 1e−3)
Renal failure	0.28 (0.45)	0.29 (0.45)	0.07 (0.25)	− 0.490 (*P* = 0.625)	18.517 (*P* < 1e−3)	20.819 (*P* < 1e−3)
Hypothyroidism	0.15 (0.36)	0.13 (0.34)	0.11 (0.31)	1.662 (*P* = 0.097)	4.063 (*P* < 1e−3)	2.642 (*P* = 0.008)
Depression	0.12 (0.32)	0.13 (0.34)	0.14 (0.35)	− 1.093 (*P* = 0.275)	− 2.016 (*P* = 0.0439)	1.055 (*P* = 0.291)
Valvular disease	0.12 (0.32)	0.13 (0.34)	0.07 (0.26)	− 0.993 (*P* = 0.321)	4.668 (*P* < 1e−3)	6.549 (*P* < 1e−3)
Peripheral vascular	0.11 (0.32)	0.12 (0.33)	0.05 (0.23)	− 0.602 (*P* = 0.547)	6.533 (*P* < 1e−3)	8.076 (*P* < 1e−3)

*P* value smaller than 0.0001 are shown in scientific notation; Missing values in clinical characteristics were dropped in T Test

## Appendix 4

See Fig. 8.

## Appendix 5

See Fig. 9.

## Appendix 6

See Fig. 10.

## Appendix 7

See Fig. 11.

## Abbreviations

SANMF: Subgraph-augmented non-negative matrix factorization
MIMIC-III: Medical information mart for intensive care III
ICU: Intensive care unit
SOFA: Sequential organ failure assessment score
EGDT: Early goal-directed therapy
SIRS: Systemic inflammatory response syndrome
NMF: Non-negative matrix factorization
EHRs: Electronic health records
Diastolic BP: Diastolic blood pressure
Systolic BP: Systolic blood pressure
Ph: Potential of hydrogen
GCS: Glasgow coma scale
SaO_2_: Blood oxygen saturation
FiO_2_: Fraction of inspired O_2_
PaO_2_: Partial pressure of arterial oxygen
PTT: Partial prothrombin time
INR: International normalized ratio
AST: Aspartate aminotransferase
CO_2_: Carbon dioxide
PAM: Partitioning around medoids
